# Aspirator in Retention of Urine

**Published:** 1880-03

**Authors:** W. O. Roberts


### ASPIRATOR IN RETENTION OF URINE.

By W. O. ROBERTS, M.D.

In the British Medical Journal of January 31, 1880, Mr. C. Burgoyre Pasley, Surgeon-in-charge Colonial Hospital, Trinidad, thus enumerates the advantages of the use of the aspirator in the treatment of retention of urine due to organic stricture with spasm superadded:

1. The operation is very simple, and, if skillfully performed, absolutely without risk.

2. The pain caused by the introduction of the trocar is trifling and momentary.

3. Patient is afforded instantaneous relief.

4. In every case the patient pass.d urine freely in a few hours.

5. The delay under the usual method of treatment is avoided, and patient is saved much unnecessary suffering.

And he adds: "Might not the laceration of the urethra commonly seen as a result of the rough and unskillful handling of catheters be avoided by resorting more frequently to the easy and perfectly safe operation of aspiration?"

The question leads me to record the following cases of retention, three of which were due to organic stricture and two to enlarged prostrate, in which I used the aspirator:

I.—S. P., aged twenty-eight, had suffered for several years from organic stricture. After exposure to a cold rain one afternoon he had great difficulty that night in voiding his urine, and the following morning had complete retention. At noon of the same day, when I saw him, his sufferings were intense. Attempts at the introduction of catheters had produced false passages, with oozing of blood from the meatus. I advised the immediate use of the aspirator; and with that instrument, the needle entering the bladder through the abdominal walls just above the pubes, drew off twenty-four ounces of urine. No further attempts at catheterization were made for three days, the urine during this time being drawn off twice daily with the aspirator, when I succeeded without difficulty in passing a No. 6 gum instrument. No trouble followed the aspirations.

II.—Saw, with Drs. Cowling and McEvoy, a negro man, aged seventy, who had suffered from great difficulty in passing urine for several days, but had had complete retention for thirty-six hours. The bladder formed a tumor in the abdominal cavity the size of a cocoanut. There was great general prostration. All attempts at catheterization proving fruitless, the aspirator was used, and about a quart of dark, bloody-colored and fetid urine drawn off. Patient died of uremic poisoning in eight hours after the operation.

III.—G. H., aged thirty, came to my office at 8 o'clock, P.M., to be relieved of a distended bladder. He stated that he had been the subject of organic stricture for five years; that whenever he was exposed to severe cold or drank to excess he would have retention of urine; that he was generally able to relieve himself with a No. 6 gum catheter, but that this time he had utterly failed. He had been visiting the beer-gardens all the afternoon, and drank very freely. Finding upon examination that in his attempts to pass the catheter he had greatly lacerated his urethra, I immediately used the aspirator and drew off about twenty ounces of urine; then directed the patient to go home and keep warm in bed with hot applications to his perineum. The following morning he was able to void urine as well as usual, and I saw nothing more of the case.

IV.—Saw, with Dr. McDonough, a case of retention of urine due to enlarged prostate in a man sixty-eight years of age. We were told that the patient had suffered with difficulty in passing his urine for a long time; that about a year previously he had retention for twenty-four hours, which was relieved by a catheter; that he had not passed urine for forty-eight hours, when they sent for Dr. McD. The doctor found the bladder unusually distended, rising as high as the umbilicus. Failing to introduce a catheter, he sent for me to aspirate. I drew off one hundred and twenty-six ounces of urine. I was unable to see the case again until the following morning, twenty-four hours from date of last visit, when we got seventy ounces. We were to have seen him again that evening, but learned that he had died at 2 P.M., his death being a very quiet one.

V.—Was asked by Dr. L. P. Yandell to see Mr. C. H., aged forty-five, who was suffering intensely from retention of urine. Had had organic stricture since a young man, but never before suffered from retention. He was in the last stages of consumption, and unable to lie down on account of excessive coughing and a feeling of suffocation. By his own attempts at catheterization in the sitting posture he had produced laceration of the uretha, followed by considerable hemorrhage. By soaking the penis in hot water he succeeded in voiding a small quantity of urine. Notwithstanding this fact, I insisted upon the use of the aspirator, but he persistently refused, giving as his reason that he believed he would be able to "soak it" all out during the night, and that he would rather "die in the attempt" than have the needle stuck into his belly. Early the following morning, however, I was called in great haste, and on my arrival found him in such agony that he immediately consented to aspiration. When the needle was introduced he remarked that it caused much less suffering than the attempts at catheterization had done. Twenty-three ounces of urine were drawn off, to the patient's great relief. This case is still under treatment, and has been aspirated so far six times.

Dr. Dieulafoy, in his work on the aspirator, published in 1873, reports eighteen cases of retention due to various causes—viz: organic and traumatic stricture, cancer, prostatic enlargement, etc.—in which the operation was performed—in eight cases once, two twice, one three, one four, one six, one eight, one eleven, one fourteen, one twenty-three, and one "many times"—with only four deaths. One of these was due to dysentery, one to uremia, one to peritonitis the result of an injury, and one to malignant disease. In the case that died of dysentery the post mortem showed that the "bladder was slightly congested on the internal surface. The vesical mucous membranes at the points of puncture showed four very small blackish marks, but there was no abscess and no sanguine infiltration into the vesical walls. The anterior part of the bladder had contracted no adhesions with the corresponding abdominal walls. The prevesical cellular tissue showed here and there in its substance several indurated points, which were found again in the subperitoneal cellular tissue of the abdominal walls. The coloration was evidently the result of slight infiltration of blood, which had taken place at different times. There were, however, no sanguinous collections and no abscess." This case had been aspirated twenty-three times.

In the case of cancer eleven aspirations were made just above the pubes, within a space not larger than a half-franc piece, without provoking the least inflammation in the peritoneum or bladder. A post mortem examination unfortunately was refused by the family in my fatal cases.

